# Prenatal stress programs neuroendocrine stress responses and affective behaviors in second generation rats in a sex-dependent manner

**DOI:** 10.1016/j.psyneuen.2015.08.010

**Published:** 2015-12

**Authors:** Natalia J. Grundwald, Paula J. Brunton

**Affiliations:** The Roslin Institute and R (D) SVS, University of Edinburgh, Easter Bush Campus, Midlothian, EH25 9RG, UK

**Keywords:** Anxiety, Glucocorticoids, HPA axis, Prenatal stress, Sex differences, Trans-generational

## Abstract

An adverse environment in early life is often associated with dysregulation of the hypothalamo-pituitary-adrenal (HPA) axis and higher rates of mood disorders in adulthood. In rats, exposure to social stress during pregnancy results in hyperactive HPA axis responses to stress in the adult offspring and heightened anxiety behavior in the males, but not the females. Here we tested whether, without further intervention, the effects of prenatal stress (PNS) in the first filial generation (F1) are transmitted to the F2 generation via the maternal line. F1 control and PNS female rats were mated with control males and housed under non-stress conditions throughout pregnancy. HPA axis responses to acute stress, anxiety- and depressive-like behavior were assessed in the adult F2 offspring.

ACTH and corticosterone responses to an acute stressor were markedly enhanced in F2 PNS females compared with controls. This was associated with greater corticotropin releasing hormone (*Crh*) mRNA expression in the paraventricular nucleus and reduced hippocampal glucocorticoid (*Gr*) and mineralocorticoid receptor (*Mr*) mRNA expression. Conversely, in the F2 PNS males, HPA axis responses to acute stress were attenuated and hippocampal *Gr* mRNA expression was greater compared with controls.

F2 PNS males exhibited heightened anxiety-like behavior (light-dark box and elevated plus maze) compared with F2 control males. Anxiety-like behavior did not differ between F2 control and PNS females during metestrus/diestrus, however at proestrus/estrus, F2 control females displayed a reduction in anxiety-like behavior, but this effect was not observed in the F2 PNS females. Heightened anxiety in the F2 PNS males was associated with greater *Crh* mRNA expression in the central nucleus of the amygdala compared with controls. Moreover, Crh receptor-1 (*Crhr1*) mRNA expression was significantly increased, whereas *Crhr2* mRNA was significantly decreased in discrete regions of the amygdala in F2 PNS males compared with controls, with no differences in the F2 females. No differences in depressive-like behavior (sucrose preference or forced swim test) were observed in either sex. In conclusion, the effects of maternal stress during pregnancy on HPA axis regulation and anxiety-like behavior can be transmitted to future generations in a sex-dependent manner. These data have implications for human neuropsychiatric disorders with developmental origins.

## Highlights

• Stress responses are greater in prenatally stressed F2 generation female offspring.
• Stress responses are attenuated in prenatally stressed F2 generation male offspring.
• Altered stress responses may result from changes in glucocorticoid feedback.
• Prenatal stress increases anxiety in the F2 generation male offspring.
• Heightened anxiety is associated with altered gene expression in the amygdala.

## 1 Introduction

Maternal exposure to stress during pregnancy can have wide-ranging and long-lasting effects on the offspring’s brain and behavior. A growing body of evidence supports the hypothesis that some psychiatric and behavioral disorders in humans have developmental origins (Glover, 2015, King et al., 2012). The phenomenon of ‘fetal programming’ of the brain by prenatal stress is well established in rodents and is generally associated with heightened anxiety- and depressive-like behaviors and augmented stress responses (Abe et al., 2007, Brunton and Russell, 2010, Mueller and Bale, 2008, Vallee et al., 1997), though some effects are evidently sex-dependent (for review see (Brunton, 2010, Weinstock, 2007)). The neuroendocrine stress axis, the hypothalamo-pituitary-adrenal (HPA) axis, is particularly susceptible to fetal programming by prenatal stress (Maccari et al., 2014) and the resultant HPA axis dysfunction may underpin altered affective traits and an increased propensity for developing psychiatric disorders (Wingenfeld and Wolf, 2011).

Using an ethologically relevant rodent model of social stress (i.e. resident-intruder test) in pregnancy, we have previously shown that both male and female prenatally stressed offspring display markedly enhanced HPA axis responses to acute physical and psychological stressors in later life (Brunton and Russell, 2010), reflected by greater stress-induced ACTH and corticosterone secretion and greater levels of corticotropin-releasing hormone (*Crh*) mRNA expression in the medial parvocellular division of the paraventricular nucleus (mpPVN) (Brunton and Russell, 2010). Impaired central glucocorticoid negative feedback regulation of the HPA axis may explain enhanced HPA axis responses to stress in prenatally stressed offspring and is supported by findings of reduced hippocampal expression of mRNA for mineralocorticoid receptor (*Mr*) in our model or both glucocorticoid receptor (*Gr*) and *Mr* in other models of prenatal stress (Maccari et al., 2014).

In addition, we have demonstrated heightened anxiety-like behavior on the elevated plus maze in the adult male, but not the female prenatally stressed offspring (Brunton and Russell, 2010). The central CRH system plays a major role in the regulation of anxiety-like behavior, particularly at the level of the amygdala (Schulkin, 2006). In rodent models, increased anxiety-related behavior is associated with greater *Crh* gene expression in the amygdala and central administration of CRH is able to induce anxiety-like behavior in rats (Dunn and Berridge, 1990, Schulkin, 2006). Moreover in prenatally stressed rats, the anxious phenotype is associated with greater *Crh* mRNA expression, CRH content and CRH release in the amygdala (Brunton and Russell, 2010, Cratty et al., 1995, Zohar and Weinstock, 2011).

CRH exerts its actions through two receptors: CRH-type 1 (CRHR1) and CRH-type 2 (CRHR2), with both receptors present in the amygdala, but with distinct expression profiles (Van Pett et al., 2000). Several lines of evidence from studies using receptor antagonists, antisense oligonucleotides and knockout mice strongly support a role for CRHR1 in mediating the anxiogenic effects of CRH (Liebsch et al., 1995, Muller et al., 2003, Smith et al., 1998, Wang et al., 2012). The role for CRHR2 in regulating anxiety-like behavior is less clear (Bale and Vale, 2004), however several studies support an anxiolytic role for CRHR2 (Bale et al., 2000, Kishimoto et al., 2000, Vetter et al., 2002). In our rat model of prenatal stress, we have demonstrated that increased anxiety-like behavior in the adult male offspring is associated with increased *Crhr1* and decreased *Crhr2* mRNA expression in the amygdala (Brunton et al., 2011). These data are consistent with other rodent models of early life stress where an anxious phenotype has been identified (Wang et al., 2013, Wang et al., 2012, Zohar and Weinstock, 2011).

A large body of data supports the hypothesis that the long-term effects of prenatal stress on the HPA axis and behavior involve stable and persistent changes in gene function. The underlying mechanisms by which an adverse prenatal environment is embedded in the genome have yet to be fully elucidated however epigenetic modifications are likely to play a key role (Bale, 2015, Maccari et al., 2014). This raises the possibility of transmission of prenatal stress effects to future generations. It has become increasingly evident that the adverse effects of a sub-optimal environment in early life can be transmitted to subsequent generations, apparently via non-genomic mechanisms (Bale, 2015). Most extensively studied is maternal malnutrition (under- or over-nutrition) during pregnancy. In guinea pigs, the effects of maternal under-nutrition during pregnancy on HPA axis activity in the F1 offspring is transmitted to the F2 generation (Bertram et al., 2008) and in mice, phenotypes resulting from maternal high-fat diet (e.g. insulin insensitivity) can be passed to subsequent generations (Dunn and Bale, 2011).

Studies where pregnant rodents have been administered synthetic glucocorticoids (e.g. dexamethasone or betamethasone) to mimic maternal stress exposure have demonstrated changes in glucose tolerance, HPA axis regulation and anxiety-like behavior in the second generation offspring (Drake et al., 2005, Iqbal et al., 2012), however to date, very few studies have specifically investigated transgenerational inheritance of phenotypes induced by prenatal stress exposure. Thus, the aim of the current study was to investigate whether, without further intervention, the effects of social stress exposure during pregnancy on HPA axis function and related behaviors in the F1 offspring are transmitted to the F2 offspring and whether there were any sex differences in transmission.

## 2 Materials and methods

### 2.1 Animals

Sprague-Dawley rats for the parental (P) generation were purchased from Charles River (Margate, Kent, UK). The first (F1) and second (F2) filial generations of control and prenatally stressed (PNS) offspring were bred in-house. Rats were maintained on a 12–12 h light–dark cycle, under controlled temperature (22 ± 2°C) and humidity (55 ± 5%) with free access to standard 14% protein rodent diet (Harlan Teklad). Breeding females were ad libitum fed a 50:50 mixture of 14% and 19% protein diet (Harlan Teklad) throughout pregnancy and lactation. Rats were group housed (4–6 females, 3–5 males) in open-top cages. A maximum of 2 rats/sex from each F2 litter were used/group for each experiment. All experiments were approved by the local Animal Welfare and Ethical Review Body and performed in accordance with the UK Animals (Scientific Procedures) Act 1986 and the European Directive (2010/63/EU).

#### 2.1.1 Mating and pregnancy monitoring

Pairs of rats from the P or F1 generations were mated overnight. The presence of a semen plug in the breeding cage was designated day 1 of pregnancy. All males used for breeding were non-stressed controls. To generate the F1 offspring, pregnant females were either undisturbed throughout pregnancy (producing F1 control offspring) or exposed to repeated social stress (see Section 2.2) to produce PNS F1 offspring. To generate the F2 offspring, adult unmanipulated F1 females born to either control mothers or born to mothers that experienced social stress during pregnancy (see Section 2.2) were mated with control males. All the pregnant F1 rats were undisturbed throughout pregnancy, except for weighing every 4 days to monitor pregnancy progression. Pregnant rats were initially group housed until day 14 (P) or 20 (F1) of pregnancy, after which time they were separated into single cages. Soon after parturition, litter sizes, pup sex and pup weights were recorded. Pups were weighed again on post-natal day (PND) 8. Dams remained with their litters until weaning at PND 23 (F1) or 25 (F2), then offspring were housed in groups by litter and sex under standard conditions (see Section 2.1) until experiments commenced.

### 2.2 Prenatal stress paradigm

Pregnant rats (10–12 weeks old) forming the P generation (‘grandmothers’) were exposed to repeated social stress utilizing a resident-intruder paradigm as previously described (Brunton and Russell, 2010). Briefly, pregnant rats were placed in the cage of a different lactating ‘resident’ rat (days 4–9 of lactation) for 10 min/day on days 16–20 of pregnancy. We have previously demonstrated this social stress paradigm increases corticosterone secretion in the pregnant rats (Brunton and Russell, 2010). Pregnant control females (forming the P1 generation) remained in their home cages throughout gestation except for weighing on days 16 to 20.

### 2.3 Surgery: Jugular vein cannulation

Adult F2 control and PNS rats (males aged 13–15 weeks and females 16–17 weeks) were fitted with a silicone jugular vein cannula filled with sterile heparinized 0.9% saline (50U heparin/ml) 3 days prior to blood sampling under 2–3% isofluorane anesthesia in 1200 ml/min oxygen using aseptic technique, as previously described (Brunton and Russell, 2010). Following surgery rats were individually housed.

### 2.4 Blood sampling and stress exposure

Between 08.00 and 09.00 h on the day of blood sampling, the i.v. cannulae were connected to PVC tubing filled with sterile heparinized saline connected to a 1 ml syringe. 90 min later, blood sampling commenced to assess HPA axis responses to an acute physical stressor (systemic interleukin-1β; IL-1β) or psychological stressor (restraint). A basal blood sample (0.25 ml) was withdrawn, then rats were either (i) given no treatment (non-stress groups), (ii) administered 500 ng/kg of recombinant human IL-1β i.v. (R&D Systems Europe Ltd., Abingdon, UK), or (iii) restrained for 30 min in a transparent rodent restrainer (Stoelting Europe, Dublin, Ireland). Further blood samples (0.25 ml) were collected 15, 30, 60, 90 and 120 min after the onset of stress. Blood was collected in 5% (w/v) EDTA then centrifuged at 4 °C to separate the plasma. Withdrawn blood was immediately replaced with an equivalent volume of sterile 0.9% saline. Plasma was stored at −20 °C until assayed for ACTH and corticosterone. 4 h after stress onset (the optimum time for detecting changes in *Crh* and *Avp* mRNA following restraint or IL-1β (Harbuz et al., 1994, Harbuz and Lightman, 1989, Harbuz et al., 1992)), rats were killed by conscious decapitation. Brains were rapidly removed, frozen on dry ice then stored at −75 °C until cryosectioning.

### 2.5 *In situ* hybridization (ISH)

To test whether differences in ACTH and corticosterone responses to stress were a reflection of altered drive from the mpPVN neurons, ISH for *Crh* and arginine vasopressin (*Avp*; acts synergistically with CRH as a co-secretagogue of ACTH) mRNA was performed. Moreover, to establish whether the capacity for glucocorticoid-mediated negative feedback control of the HPA axis was altered in F2 PNS rats, hippocampal *Gr* and *Mr* mRNA expression was analyzed. Finally, to establish whether differences in anxiety-like behavior were associated with changes in gene expression in the amygdala, we quantified *Crhr1* and *Crhr2* mRNA expression.

#### 2.5.1 Brain sectioning and probes

15 μm coronal cryostat sections were collected onto Polysine™ coated slides (4 sections/slide) and stored at −75 °C until processing. For detection of *Avp* mRNA in the mpPVN, a 3′ end-^35^S-radio-labelled oligonucleotide probe (5′-GAC-CCG-GGG-CTT-GGC-AGA-ATC-CAC-GGA-CTC-TTG-TGT-3′) complimentary to rat *Avp* mRNA (sequence ID: X01637.1) was used (Brunton and Russell, 2010).

^35^S-UTP radio-labelled cRNA sense and antisense probes were synthesized and used to detect *Crhr1* mRNA in the central nucleus of the amygdala (CeA), basolateral amygdala (BLA), medial amygdala (MeA) and the basomedial amygdala (BMA)*, Crh-r2* mRNA in the BMA and MeA and *Gr* and *Mr* mRNA in the hippocampus, as previously described (Brunton et al., 2011, Brunton and Russell, 2010). To detect rat *Crh* mRNA in the mpPVN and the CeA, ^35^S-UTP labelled riboprobes were synthesized from the linearized pBluescript vector expressing a 518 bp cDNA fragment encoding rat *Crh*. The plasmid was linearized with Hind III and XbaI and transcribed from the T7 and T3 promoters to synthesize the sense and antisense cRNA probes, respectively. ISH was performed as previously described (Brunton et al., 2011, Brunton and Russell, 2010). In each case, following overnight hybridization and post-hybridization washes, slides were dipped in liquid autoradiographic emulsion (Ilford K5, Calumet, Edinburgh, UK) and exposed at 4 °C for 4 weeks. Sections hybridized with sense probes served as negative controls and showed no signal above background.

#### 2.5.2 Quantitative analysis

The area of each brain region of interest and the overlying grain area was measured using Image J v1.46 h software (NIH, Maryland, USA) and is expressed as grain area/brain area (mm^2/^mm^2^). Background measurements were made over an adjacent area (with no specific hybridization) and subtracted. Bilateral measurements were made from 4 sections/rat. Group means were calculated from average values/rat.

### 2.6 Radioimmunoassays

Plasma ACTH and corticosterone concentrations were determined using commercially available radioimmunoassay kits (ACTH: DIAsource ImmunoAssays S.A., Belgium; sensitivity: 1.16pg/ml, intra-assay variation: 3–6.4%; corticosterone: MP Biomedicals, UK, sensitivity: 7.7 ng/ml, intra-assay variation 4.4–10.3%). For ACTH, male and female samples were measured together and for corticosterone, samples for each sex were processed in separate assays.

### 2.7 Behavioral testing

Assessment of anxiety- and depressive-like behavior was conducted in a separate set of rats from those that underwent surgery and blood sampling (i.e. the siblings of those used to assess HPA axis function). Rats were moved to the behavioral room at least 1 day prior to the experiment and testing was performed between 09:00 and 13:00 h.

#### 2.7.1 Anxiety-like behavior

Anxiety-like behavior was assessed in male and female F2 control and F2 PNS rats at ca. 9–10 weeks of age (*n* = 20 males and 40 females). As estrus cycle stage can influence anxiety-like behavior (Frye et al., 2008, Mora et al., 1996), daily vaginal smears were performed in the week preceding the experiment to predict estrus cycle stage and confirmed by smears taken immediately following the anxiety tests. Females were characterized as either being in metestrus/diestrus (*n* = 20) or proestrus/estrus (*n* = 20). Rats were exposed to each anxiety test only once.

##### 2.7.1.1 Light-dark box

The light-dark box (LDB)(Crawley, 1981) consisted of 1 transparent box (217 lux) and 1 black box (12 lux) with a lid (both 40 × 40 × 40 cm) positioned under an infrared camera. Rats were placed in the dark compartment and recorded for 5 min using Ethovision XT software (Noldus, Wageningen, The Netherlands). The time spent in each compartment was measured.

##### 2.7.1.2 Elevated plus maze

Two days after the LDB test, the same rats were tested on the elevated plus maze (EPM). The EPM consisted of 2 open arms (100 × 10 cm each; 337 lux) which intersected at 90° to form a plus shape with 2 closed arms with black walls (<100 lux) elevated 50 cm above the floor (Pellow et al., 1985). An infrared camera recorded behavior and the data was analyzed with Ethovision XT. Rats were placed at the intersection of the 4 arms facing an open arm and allowed to freely explore for 10 min. The time spent in each arm was recorded.

In each case, the rat was deemed to be in a specific arm/compartment when all 3 points (nose point, center point and tail base) were in the zone. All apparatus was cleaned with 70% ethanol and allowed to air dry for ≥5 min before each trial.

#### 2.7.2 Depressive-like behavior

To test for the presence of a depressive-like phenotype, rats were tested for two hallmark traits: anhedonia and behavioral despair. The same set of rats that underwent testing on the EPM and LDB were used.

##### 2.7.2.1 Sucrose preference test

To test for anhedonia, the sucrose preference test (Katz, 1982, Pryce et al., 2005) was performed at 18 weeks of age. Rats were housed in same-sex sibling pairs (to control for the potential confounding factor of social isolation) with two drinking bottles/cage. Two additional naïve F2 control males (i.e. that had not undergone anxiety testing) were included. There was no difference in any parameter between the rats that had previously been exposed to the EPM and LDB and these naïve rats, so data was pooled. On day 1, rats were given *ad libitum* access to food and water for 24 h. On days 2 and 3 rats were allowed free access to food but were given a choice of either water or 2% sucrose solution. Intake was calculated by weighing the bottles at the same time each day. On day 2, the sucrose bottle was randomly positioned on the left or right side of the cage and swapped on day 3 to control for any side preference. Data are reported as mean intake per cage and statistics were performed on the data per caged pair (not per individual rat).

##### 2.7.2.2 Forced swim test

Five weeks after the sucrose preference test (i.e. at 23 weeks of age) rats were tested for behavioral despair using a modified Porsolt forced swim test (FST)(Detke et al., 1995). The experiment was performed over 2 days. On day 1, rats were forced to swim in a glass cylinder filled to a depth of 30 cm with tap water (23–25 °C) for 10 min. The following day they were placed in the same cylinder for 5 min and their behaviors were video-recorded. Time spent swimming, climbing and floating (an indicator of despair) was measured using Observer 5 (Noldus).

### 2.8 Statistical analysis

Statistical tests were performed using Sigmaplot 11.0 (Systat Software Inc., London, UK). For analysis of the ACTH and corticosterone data a two-way repeated measures (RM) ANOVA followed by Student Newman Keuls (SNK) pairwise comparison tests were used. For analysis of *Crh* and *Avp* mRNA data, a two-way ANOVA followed by SNK pairwise comparison tests were used. To analyze the effect of estrus cycle stage and PNS history on anxiety and data from the depressive-like behavior tests, a two-way ANOVA followed by SNK pairwise comparison tests were used. Simple group comparisons between two groups (i.e. F2 controls and F2 PNS rats) were performed using either a Student's t-test (for normally distributed data) or Mann–Whitney-*U* test for data that was not normally distributed. Results are reported as group means ± SEM or group medians with the interquartile range (IQR: quartiles 25–75) for non-normally distributed data. In each case, *p* ≤ 0.05 indicates a significant difference.

## 3 Results

### 3.1 Pregnancy and litter characteristics

Social stress did not affect pregnancy weight gain (day 1–20) in the P generation (control: 58.9 ± 4%, stressed: 55.4 ± 2.6%). Body weight in the F1 females was similar at the start of pregnancy (F1 control: 291 ± 8 g, F1 PNS: 306 ± 8 g), as was weight gain from pregnancy day 1-20 (F1 control: 38.3 ± 1.5%, F1 PNS: 39.1 ± 2.4%). The median litter size (F1 control: 15.5 (IQR 15-17), F1 PNS: 15.5 (15-16.75)), mean pup birth weight (F1 control: 5.96 ± 0.12 g, F1 PNS: 6.06 ± 0.2 g) and male to female ratio (F1 control: 0.87 ± 0.18, F1 PNS: 0.74 ± 0.19) did not differ between the groups in the F1 generation. There were no significant differences between the F2 litters (Table 1).

### 3.2 HPA axis responses to acute stress in second generation control and PNS rats

#### 3.2.1 ACTH and corticosterone secretion

##### 3.2.1.1 Females

**Fig. 1:**
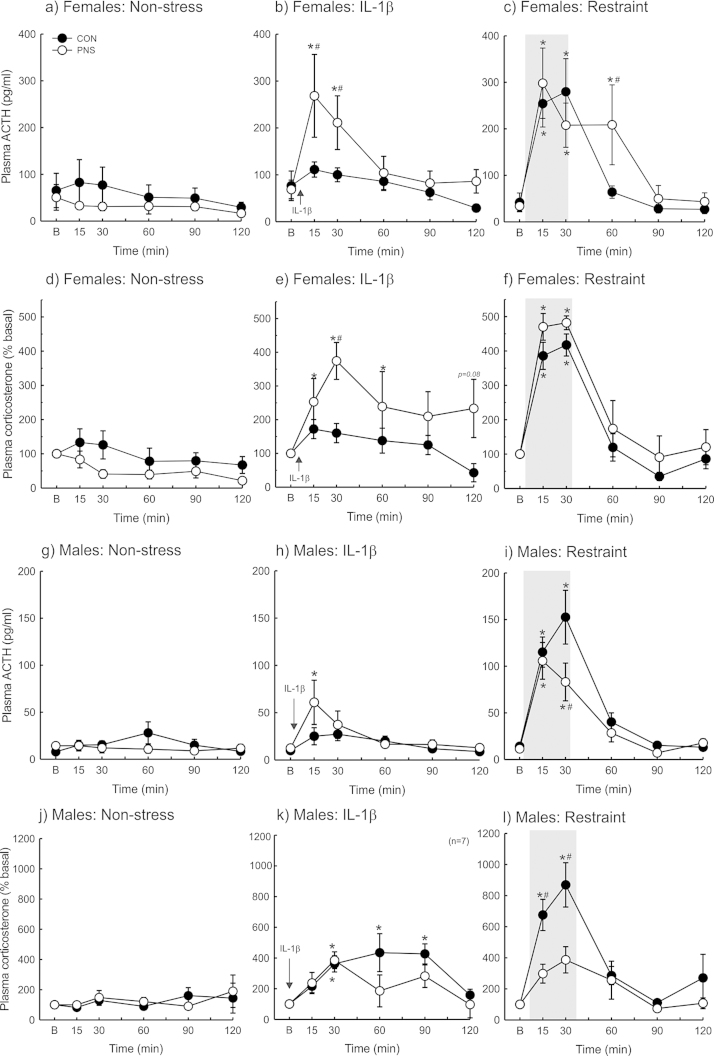
Effect of acute stress exposure on ACTH and corticosterone secretion in second generation control and PNS rats. Age-matched second generation control (CON; filled circles) and PNS (open circles) male and female rats were blood sampled under non-stress (ns) conditions or before and after administration of intravenous interleukin-1β (IL-1β; 500ng/kg) or exposure to 30 min restraint (grey bars). A basal (B) blood sample was collected (at *t* = −1 min). At *t* = 0 min rats remained undisturbed in their home cage (non-stress), were placed in a rodent restraint tube for 30 min or were administered IL-1β. Further blood samples were collected at *t* = 15, 30, 60, 90 and 120 min. Plasma ACTH concentrations in female rats in the (a) non-stress; (b) IL-1β-treated; and (**c**) restraint groups. *p < 0.05 vs B values in the same group; #*p* < 0.04 vs the other group at the same time point (two-way RM ANOVA with SNK). The increase in plasma corticosterone concentrations from basal levels in female rats in the (d) non-stress; (e) IL-1β-treated; and (f) restraint groups. *p < 0.01 vs B values in the same group; #*p* < 0.02 vs the other group at the same time point (two-way RM ANOVA with SNK). Plasma ACTH concentrations in male rats in the (g) non-stress; (h) IL-1β-treated; and (i) restraint groups. *p < 0.002 vs B values in the same group; #*p* < 0.001 vs the other group at the same time point (two-way RM ANOVA with SNK). The increase in plasma corticosterone concentrations from basal levels in female rats in the (j) non-stress; (k) IL-1β-treated; and (l) restraint groups. *p < 0.01 vs B values in the same group; #*p* < 0.005 vs the other group at the same time point (two-way RM ANOVA with SNK). Rat numbers/group are *n* = 5–7 for females and *n* = 6–7 for males. Data are group means ± SEM.

There was no difference in basal plasma ACTH concentrations between female F2 control and F2 PNS rats (Fig. 1a–c) and no significant change in ACTH secretion in the non-stress groups (Fig. 1a). There was a significant effect of time (*F*_(5,44)_ = 10.3; *p* < 0.001, two-way RM ANOVA) and a significant group (PNS history) × time interaction (*F*_(5,44)_ = 2.7; *p* = 0.03) on plasma ACTH concentrations in the female rats after IL-1β administration (Fig. 1b). Plasma ACTH concentrations were significantly greater in the F2 PNS females than in the F2 control females 15 and 30 min after IL-1β administration (*p* < 0.02 and *p* < 0.04, respectively, SNK pairwise comparison test; Fig. 1b). There was a significant effect of time (*F*_(5,50)_ = 15.6; *p* < 0.001) on plasma ACTH concentrations in the female rats in response to restraint (Fig. 1c). Restraint significantly increased plasma ACTH concentrations to a similar extent in both the F2 control and F2 PNS females (*p* < 0.001, SNK pairwise comparison test; Fig. 1c). Plasma ACTH concentrations returned to basal levels within 60 min of the onset of restraint in the control females, however levels remained significantly greater than basal levels in the F2 PNS females (*p* = 0.025, SNK pairwise comparison test; Fig. 1c).

There was no significant difference in basal plasma corticosterone concentrations between control and F2 PNS female rats. Mean basal plasma corticosterone concentrations were 187 ± 37 ng/ml in the female F2 control rats and 165 ± 45 ng/ml in the female F2 PNS rats. There was no change in corticosterone secretion in the non-stressed groups (Fig. 1d). There was a significant effect of time (*F*_(5,53)_ = 6.7; *p* < 0.001, two-way RM ANOVA) and a significant group (PNS history) × time interaction (*F*_(5,53)_ = 2.4; *p* < 0.05) on plasma corticosterone concentrations in the female rats following IL-1β administration (Fig. 1e). The increase in corticosterone secretion was significantly greater in the F2 PNS females than in the F2 control females 30 and 60 min after IL-1β administration (*p* < 0.01, SNK pairwise comparison test; Fig. 1e), with a tendency for a prolonged response in the F2 PNS females (*p* = 0.08 at 120 min post-IL-1β, SNK pairwise comparison test; Fig. 1e). There was a significant effect of time (*F*_(5,60)_ = 54.2; *p* < 0.001) on corticosterone secretion in the female rats in response to restraint (Fig. 1f). Restraint significantly increased plasma corticosterone concentrations in both the F2 control and F2 PNS females (*p* < 0.001; Fig. 1f), however there were no significant differences between the groups (Fig. 1f).

##### 3.2.1.2 Males

There was no significant difference in basal plasma ACTH concentrations between male F2 control and F2 PNS rats (Fig. 1g–i) nor any significant change in ACTH secretion in the non-stress groups (Fig. 1g). There was a significant effect of time (*F*_(5,55)_ = 4.0; *p* = 0.003, two-way RM ANOVA) on plasma ACTH concentrations in the male rats following IL-1β administration (Fig. 1h). Plasma ACTH concentrations were significantly increased from basal levels in the F2 PNS males (*p* = 0.017, SNK pairwise comparison test) 15 min post-IL-1β administration, but not in the F2 control males (Fig. 1h) and there was no significant difference between the groups. There was a significant effect of time (*F*_(5,53)_ = 32.1; *p* < 0.001) and a significant group (PNS history) × time interaction (*F*_(5,53)_ = 2.5; *p* = 0.04) on plasma ACTH concentrations in the male rats in response to restraint (Fig. 1i). Restraint significantly increased ACTH secretion in both the F2 control and F2 PNS males (*p* < 0.001, SNK pairwise comparison test; Fig. 1i), however the peak response (at *t* = 30 min) to restraint was significantly lower in the F2 PNS males, compared with the F2 control males (*p* < 0.001, SNK pairwise comparison test; Fig. 1i).

There was no significant difference in basal plasma corticosterone concentrations between F2 control and F2 PNS male rats. Mean basal plasma corticosterone concentrations were 35.6 ± 7.3 ng/ml in the male F2 control rats and 36.0 ± 5.7 ng/ml in the male F2 PNS rats. There was no change in corticosterone secretion in the non-stressed groups (Fig. 1j). There was a significant effect of time (*F*_(5,55)_ = 8.4; *p* < 0.001, two-way RM ANOVA) on plasma corticosterone concentrations in the male rats following IL-1β administration (Fig. 1k). IL-1β significantly increased corticosterone secretion in both the F2 control and F2 PNS males within 30 min of IL-1β administration (*p* = 0.01). The IL-1β-induced increase in corticosterone secretion remained significantly greater than basal levels in the F2 control males at 60 and 90 min post-IL-1β (*p* < 0.001, SNK pairwise comparison test), but not in the F2 PNS males (Fig. 1k). There was a significant effect of time (*F*_(5,50)_ = 11.6; *p* < 0.001) and group (PNS history) (*F*_(1,50)_ = 7.9; *p* = 0.01) and a significant group (PNS history) × time interaction (*F*_(5,50)_ = 3.2; *p* = 0.01) on corticosterone secretion in the male rats in response to restraint (Fig. 1l). The restraint-induced increase in corticosterone secretion was significantly greater in the F2 control males compared with the F2 PNS males (*p* = 0.004 at 15 min and *p* < 0.001 at 30 min, SNK pairwise comparison test; Fig. 1l).

##### 3.2.1.3 Sex differences

As expected, there was a significant effect of sex on ACTH secretion in response to IL-1β (*F*_(1,50)_ = 10.3, *p* = 0.009, two-way RM ANOVA; Fig. 1b, h) and a significant effect of sex on ACTH secretion in response to restraint (*F*_(1,59)_ = 5.3, *p* = 0.04, two-way RM ANOVA; Fig. 1c, i) in the F2 control and in the F2 PNS rats (IL-1β groups: *F*_(1,49)_ = 8.7, *p* = 0.014, two-way RM ANOVA, Fig. 1b, h; restraint groups: *F*_(1,44)_ = 13.2, *p* = 0.005, two-way RM ANOVA, Fig. 1c, i), with significantly greater peak ACTH concentrations in the females than in the males in response to both stressors (*p* < 0.001, SNK pairwise comparison test).

#### 3.2.2 Crh and Avp mRNA expression in the mpPVN

##### 3.2.2.1 Females

**Fig. 2:**
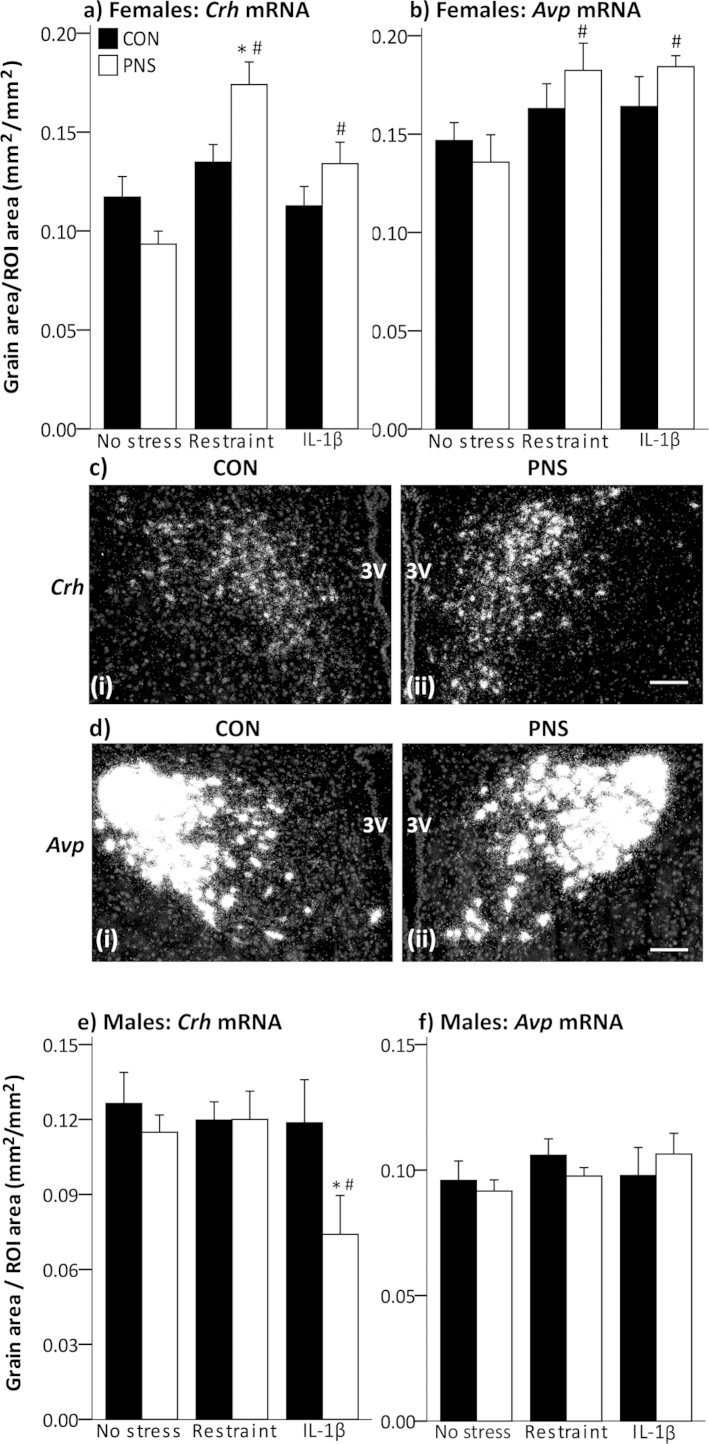
Effect of acute stress exposure on *Crh* and *Avp* mRNA expression in the mpPVN in second generation control and PNS rats. Age-matched F2 control (CON, filled bars) and F2 PNS (PNS, open bars) rats were killed 4 h after the onset of acute stress and brains were processed by *in situ* hybridization. Quantification of *Crh* mRNA expression in the medial parvocellular paraventricular nucleus (mpPVN) in (a) females and (e) males. #*p* < 0.02 versus respective non-stress group; *p < 0.02 versus respective F2 control group (two-way ANOVA with SNK). Quantification of *Avp* mRNA expression in the mpPVN in the (b) females and (f) males. Rat numbers/group are *n* = 5–7 for females and *n* = 5–11 for males. Representative dark-field images of (c) *Crh* mRNA and (d) *Avp* mRNA expression in the PVN of (i) F2 control female and (ii) F2 PNS female 4 h after restraint. Scale bar = 100 μm; 3 V, 3rd ventricle; ROI, region of interest. Data are group means ± SEM.

There was a significant effect of treatment (i.e. acute stress exposure) (*F*_(2,25)_ = 12.98, *p* < 0.001, two-way ANOVA) and a group × treatment interaction (*F*_(2,25)_ = 5.2, *p* = 0.013, two-way ANOVA) on *Crh* mRNA expression in the mpPVN in the females. *Crh* mRNA expression was significantly greater after acute stress in the F2 PNS females (restraint, *p* < 0.001; IL-1β, *p* = 0.008, SNK pairwise comparison test; Fig. 2a) compared with the non-stressed group, but not in the F2 control females. *Crh* mRNA in the mpPVN 4 h after restraint, was significantly greater in the F2 PNS females compared with the F2 control females (*p* = 0.006, SNK pairwise comparison test; Fig. 2a, c). There was a significant effect of treatment (i.e. acute stress exposure) on *Avp* mRNA expression in the mpPVN (*F*_(2,29)_ = 4.28, *p* = 0.024, two-way ANOVA) with significantly greater levels after restraint (*p* = 0.014, SNK pairwise comparison test) and IL-1β (*p* = 0.014, SNK pairwise comparison test) in the F2 PNS rats, but not in the F2 control rats, compared with the respective non-stressed rats (Fig. 2b, d).

##### 3.2.2.2 Males

There was a significant effect of PNS history (F_(1,39)_ = 4.40, *p* = 0.04, two-way ANOVA) on *Crh* mRNA expression in the mpPVN in the male F2 offspring. After IL-1β, *Crh* mRNA expression was significantly lower in the F2 PNS males compared with the F2 controls (*p* = 0.017; SNK pairwise comparison test) and compared with levels in non-stressed F2 PNS males (*p* = 0.01; SNK pairwise comparison test; Fig. 2e) There were no significant differences detected in *Avp* mRNA expression in the males (Fig. 2f).

#### 3.2.3 *Gr* and *Mr* mRNA expression in the hippocampus

##### 3.2.3.1 Females

**Fig. 3:**
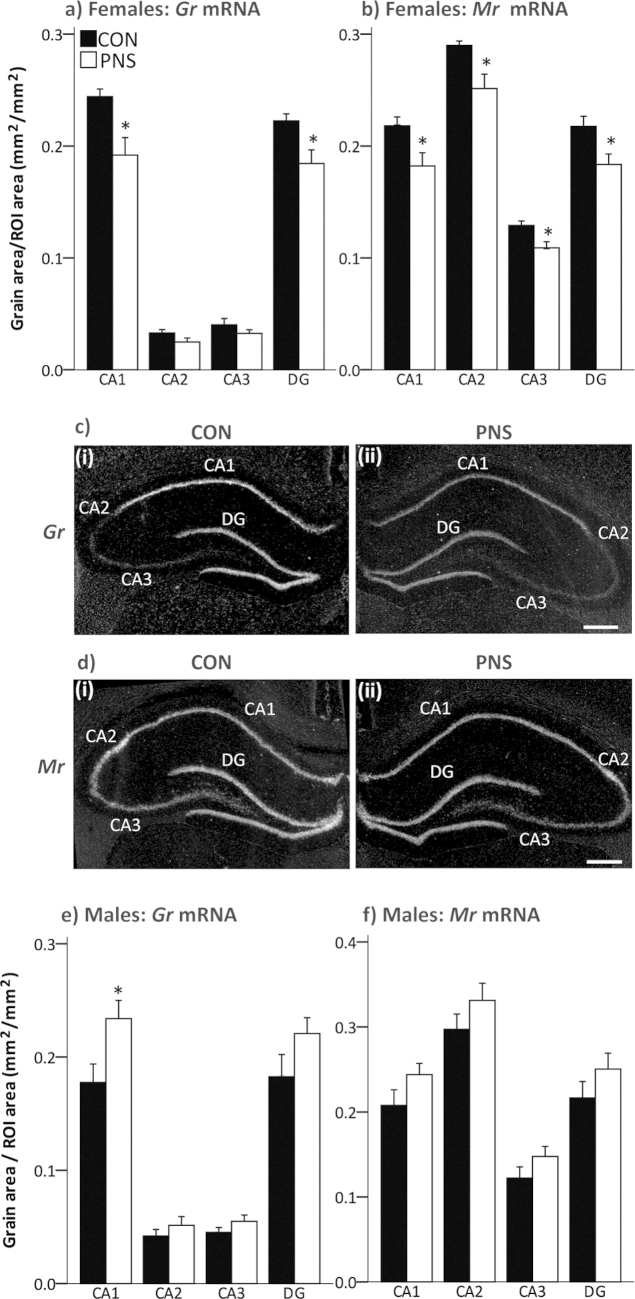
Hippocampal *Gr* and *Mr* mRNA expression under basal conditions in second generation control and PNS rats. Brains from age-matched F2 control (CON, filled bars) and F2 PNS (PNS, open bars) were processed by *in situ* hybridization. Quantification of *Gr* mRNA expression in the hippocampal subfields CA1, CA2, CA3 and dentate gyrus (DG) in (a) females and (e) males. Statistics: *p ≤ 0.03 vs F2 control group (Student’s *t*-test). Quantification of *Mr* mRNA expression in the hippocampus in (b) females and (f) males. Statistics: *p < 0.04 vs F2 control group (Student’s *t*-test). Rat numbers/group are *n* = 6 for females and *n* = 7 for males. Representative dark-field autoradiographs of basal expression of (c) *Gr* and (d) *Mr* mRNA in the hippocampus of a (i) F2 control female and (ii) F2 PNS female. Scale bar = 500 μm; ROI, region of interest.

Under basal conditions, *Gr* mRNA expression was significantly lower in the hippocampal CA1 region (t(6.9) = 3.048, *p* = 0.019) and in the dentate gyrus (DG, t(10) = 2.792, *p* = 0.019) in F2 PNS females compared with F2 controls (Fig. 3a, c; Student’s *t*-test). Moreover, expression of *Mr* mRNA was significantly lower in the CA1, (t(10) = 2.407, *p* = 0.037), CA2 (t(6.7) = 2.75, *p* = 0.03), CA3 (t(10) = 3.21, *p* = 0.009) and dentate gyrus (t(10)=2.632, p=0.025) in F2 PNS females compared with F2 control females (Fig. 3b, d; Student’s *t*-test).

##### 3.2.3.2 Males

Under basal conditions, hippocampal expression of *Gr* mRNA was significantly greater in the CA1 region in F2 PNS rats compared with the F2 controls (t(12) = −2.462, *p* = 0.03, Student’s *t*-test; Fig. 3e). No significant differences in hippocampal *Mr* mRNA expression were detected between F2 control and F2 PNS males (Fig. 3f; Student’s *t*-test).

### 3.3 Anxiety-like behavior

#### 3.3.1 Females

**Fig. 4:**
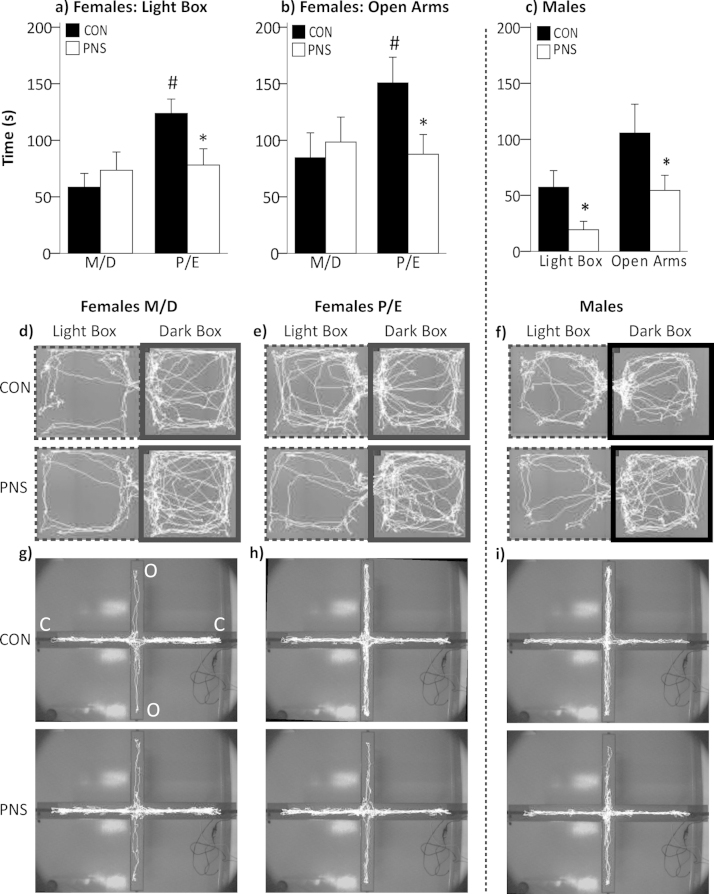
Anxiety-like behavior in the light-dark box and on the elevated plus maze. Age-matched F2 control (CON, filled bars) and F2 PNS (PNS, open bars) male and female rats were tested in the light-dark box (LDB) and 2 days later on the elevated plus maze (EPM). Females are subdivided based on estrus cycle stage. Time spent (seconds) in (a) the light compartment of the LDB and (b) the open arms of the EPM in females during metestrus/diestrus (M/D) and proestrus/estrus (P/E). Statistics: #*p *< 0.02 control M/D group; *p < 0.03 vs PNS P/E group (two-way ANOVA with SNK). (c) Time spent in the light compartment of the LDB and on the open arms of the EPM in males. Statistics: *p ≤ 0.05 vs F2 control males (Student’s *t*-test). In each case data are group means ± SEM and *n* = 10 rats/group. Representative tracking of (d) M/D female rats; (e) P/E female rats and (f) male rats in the LDB. Light compartment delineated with dashed line and dark compartment with a solid line. Representative tracking of (g) M/D female rats; (h) P/E female rats and (i) male rats on the EPM. O, open arms; C, closed arms.

There was no overall effect of PNS history (LDB: *F*_(1,36)_ = 1.21, *p* = 0.28; EPM: *F*_(1,36)_ = 1.36, *p* = 0.13, two-way ANOVA) in either of the anxiety tests in the females, however there was a significant PNS history × estrus cycle stage interaction for both the LDB (F_(1,36)_ = 4.71, *p* = 0.037; Fig. 4a, d and e) and the EPM (F_(1,36)_ = 3.3, *p* = 0.039; Fig. 4b, g and h). There was a significant increase in the time spent in the light compartment (*p* = 0.002, SNK pairwise comparison test; Fig. 4a) and on the open arms (*p* = 0.017, SNK pairwise comparison test; Fig. 4b) at proestrus/estrus, compared with metestrus/diestrus in the F2 control females, but not in the F2 PNS females (LDB: *p* = 0.814; EPM: *p* = 0.36). In addition, significantly more time was spent in the anxiogenic environments at proestrus/estrus in the F2 control females compared with the F2 PNS females (LDB: *p* = 0.027; EPM: *p* = 0.021, SNK pairwise comparison test).

#### 3.3.2 Males

F2 PNS males spent significantly less time in the light compartment of the LDB (t(13) = 2.274, *p* = 0.02, Student’s *t*-test; Fig. 4c, f) and on the open arms of the EPM (t(14) = 1.764, *p* = 0.05, Student’s *t*-test; Fig. 4c, i) compared with the F2 control males.

There was no difference in the total distance travelled in the LDB between F2 control and F2 PNS in either the males or females, indicating no effect of PNS on general mobility (data not shown).

### 3.4 CRH and CRH receptor mRNA expression in the amygdala

#### 3.4.1 Females

**Fig. 5:**
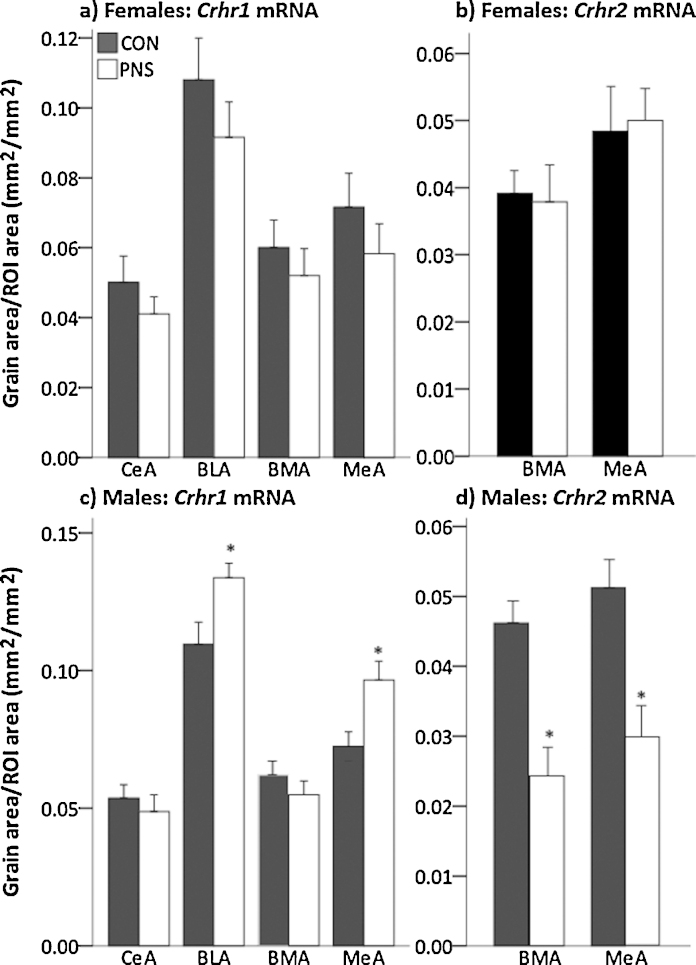
*Crhr1* and *Crhr2* mRNA expression in the amygdala under basal conditions in second generation control and PNS rats. Brains from age-matched F2 control (CON, filled bars) and F2 PNS (PNS, open bars) were processed by *in situ* hybridization. Quantification of *Crhr1* mRNA in (a) females and (c) males and *Crhr2* mRNA expression in (b) females and (d) males. Statistics: *p < 0.02 vs F2 control group (Student’s *t*-test). In each case data are group means ± SEM. Rat numbers/group are *n* = 5–6 for females and *n* = 8–9 for males. Abbreviations: BLA, basolateral amygdala; BMA, basomedial amygdala; CeA, central amygdala; MeA, medial amygdala; ROI, region of interest.

No significant differences were detected in mRNA expression for C*rh* in the CeA (F2 controls = 0.0276 ± 0.003 mm^2^/mm^2^, *n* = 6; F2 PNS = 0.0279 ± 0.003 mm^2^/mm^2^, *n* = 6), *Crhr1* in the CeA, BLA, BMA or MeA (Fig. 5a) or C*rhr2* mRNA expression in the BMA or the MeA (Fig. 5b) between F2 control and F2 PNS females under basal conditions.

#### 3.4.2 Males

*Crh* mRNA expression in the CeA was significantly greater in the F2 PNS males (0.034 ± 0.003 mm^2^/mm^2^, *n* = 12) compared with the F2 control males (0.023 ± 0.002 mm^2^/mm^2^, *n* = 12; U(23) = 122, Z = 2.89, *p* = 0.004, Mann–Whitney-*U* test) under basal conditions. C*rhr1* mRNA expression was significantly greater in the BLA (t(16) = −2.6, *p* = 0.01, Student’s *t*-test) and in the MeA (t(16) = −2.81, *p* = 0.007, Student’s *t*-test) in the F2 PNS males compared with the F2 control males (Fig. 5c), however no significant differences were detected between F2 males in *Crhr1* mRNA expression in the CeA or the BMA (Fig. 5c). Conversely, C*rhr2* mRNA expression was significantly lower in the BMA (t(14) = 4.23, *p* < 0.001, Student’s *t*-test) and in the MeA (t(14)=3.49, *p* = 0.002, Student’s *t*-test) in F2 PNS males compared with F2 control males (Fig. 5d).

### 3.5 Depressive-like behavior

#### 3.5.1 Sucrose preference

##### 3.5.1.1 Females

Although there was a significant preference for sucrose over water in both groups (*F*_(1,15)_ = 205.99, *p* < 0.001, two-way ANOVA), there was no difference in mean water (mean daily intake/pair: F2 control, 34 ± 2 g, *n* = 7 pairs; F2 PNS: 34 ± 3 g, *n* = 10 pairs) or sucrose (mean daily intake/pair: F2 control, 118 g ± 15 g; F2 PNS, 135 g ± 10 g) intake between the female F2 control and F2 PNS rats.

##### 3.5.1.2 Males

There was a significant preference for sucrose over water in the male rats (*F*_(1,9)_ = 70.942, *p* < 0.001, two-way ANOVA), however there was no difference in mean water (mean daily intake/pair: F2 control, 60 ± 5 g, *n* = 6 pairs; F2 PNS, 61 ± 4 g, *n* = 5 pairs) or sucrose (mean daily intake/pair: F2 control, 172 ± 28 g, F2 PNS, 166 ± 23 g) intake between the male F2 control and PNS rats.

#### 3.5.2 Forced Swim test (FST)

There were no group (i.e. PNS history) differences or group × behavior interactions (2-way ANOVA) between F2 control and PNS rats in the FST in either the females or the males (Table 2).

## 4 Discussion

Here we describe for the first time, sex-dependent transgenerational transmission of prenatal stress effects via the maternal line on HPA axis regulation and anxiety-like behavior in rats. Importantly, these effects occurred in the absence of any interventions in the F1 pregnancy.

Female F2 PNS rats, displayed enhanced HPA axis responses to a physical stressor (IL-1β) compared with control F2 females. The amplitude of the ACTH and corticosterone response to a psychological stressor (restraint) was similar in the F2 females, however the ACTH response was prolonged and *Crh* and *Avp* mRNA expression in the mpPVN were markedly increased in the F2 PNS females, compared with the F2 control females. Conversely, in the F2 PNS males, ACTH and corticosterone secretion in response to restraint was significantly attenuated, compared with F2 control males. Moreover, the corticosterone response to IL-1β was shorter in duration and *Crh* mRNA expression in the mpPVN was lower in the F2 PNS males, compared with the F2 controls after IL-1β treatment. These effects are in contrast to the F1 generation, in which both sexes exhibit greater HPA axis responses to stress (Brunton and Russell, 2010). The mechanisms underlying divergent sex-specific effects on HPA axis function in the F2 offspring are not known, though may involve the opposing actions of male and female gonadal steroids on HPA activity or altered neuroactive steroid generation (Brunton et al., 2015, Figueiredo et al., 2007, Seale et al., 2004, Viau and Meaney, 1996). In support, prenatal stress or glucocorticoid exposure during pregnancy is associated with altered circulating sex steroids and changes in steroid-metabolizing enzymes in the brain of the F1 offspring (Brunton et al., 2015, Dunn et al., 2010, Kapoor and Matthews, 2011). Recent studies have also indicated that maternal stress can program persistent alterations in gene expression in the placenta, which are dependent upon the sex of the fetus (Howerton et al., 2013), providing a possible route through which prenatal stress could program the fetal brain in a sex-specific manner.

Enhanced HPA axis responses to stress may result from increased excitatory drive or reduced inhibitory input to the mpPVN CRH/AVP neurons, reduced glucocorticoid negative feedback control or a combination of adaptations. Physical and psychological stressors are processed through two converging but distinct mechanisms within the brain (Herman and Cullinan, 1997). Restraint is generally processed via limbic forebrain circuits, whereas systemic IL-1β activates the HPA axis via noradrenergic brainstem inputs (Ericsson et al., 1994, Herman and Cullinan, 1997). Thus enhanced ACTH and corticosterone responses to IL-1β in the F2 PNS females may result from increased excitatory input to the mpPVN neurons by brainstem noradrenergic afferents. Likewise, attenuated ACTH and corticosterone responses to restraint in the F2 PNS males may involve reduced recruitment of PVN-signaling neurons in the forebrain.

Both MR and GR play important roles in glucocorticoid-mediated feedback control of the HPA axis following stress (Ratka et al., 1989). In the F2 PNS females, *Mr* mRNA expression was down-regulated across all of the hippocampal sub-regions and *Gr* mRNA expression was significantly lower in the CA1 and dentate gyrus. If the reduction in *Gr* and *Mr* mRNA is accompanied by reduced protein levels, these data suggest that enhanced and/or prolonged HPA axis responses to acute stress in the F2 PNS females may result from impaired glucocorticoid negative feedback control. In contrast, in the F2 PNS males *Gr* mRNA expression was increased in the CA1 hippocampal subfield which may contribute to their blunted HPA axis responses to acute stress. Indeed, increased levels of *Gr* mRNA in the hippocampal CA1 region are associated with attenuated stress-induced cortisol secretion and enhanced glucocorticoid feedback sensitivity in F2 male guinea pigs whose grandmothers were repeatedly administered the synthetic glucocorticoid, betamethasone during pregnancy (Iqbal et al., 2012).

Dysregulation of HPA axis is linked with affective disorders in humans (Gold and Chrousos, 2002, Shea et al., 2005, Young et al., 2004) and we and others have reported increased anxiety-like behavior in F1 PNS offspring (Brunton and Russell, 2010, Poltyrev et al., 2005, Vallee et al., 1997), therefore we tested F2 PNS rats for the expression of this trait using the LDB and EPM. F2 PNS males spent significantly less time in the light box and on the open arms indicating increased anxiety-like behavior. The amygdala CRH system plays a critical role in mediating anxiety behavior (Schulkin, 2006) and here the anxious phenotype in F2 PNS males was associated with significantly greater *Crh* mRNA expression in the central amygdala, consistent with findings in F1 PNS rats that also exhibit increased anxiety-related behaviors (Brunton and Russell, 2010, Cratty et al., 1995, Zohar and Weinstock, 2011). CRHR1 and CRHR2 in the amygdala play significant, though essentially opposing roles in regulating emotionality (Bale and Vale, 2004). Generally, CRHR1 activation, increases anxiety-like behavior (Koob and Thatcher-Britton, 1985; Spina et al., 2002), whereas activation of CRHR2 suppresses behavioral indicators of anxiety (Valdez et al., 2002; Bale and Vale, 2004). In accordance, *Crhr1* mRNA expression was greater in the basolateral and medial amygdala and *Crhr2* mRNA expression was lower in the basomedial and medial amygdala in the F2 PNS males compared with control males, consistent with the anxious phenotype and our previously published findings from the F1 PNS males (Brunton et al., 2011). In addition, increased hippocampal *Gr* mRNA expression is linked with anxiety in rats (Kabbaj et al., 2000), thus increased *Gr* mRNA in the F2 PNS males may also contribute to their increased anxiety-like behavior. CRH receptors within the bed nucleus of the stria terminalis (BNST) are known to mediate the effects of CRH on anxiety-like behavior (Sahuque et al., 2006). However, it is unlikely that the increased anxiety-like behavior observed in the F2 PNS males under basal conditions results from differential expression of CRH receptors in the BNST, since intra-BNST administration of specific antagonists for CRHR1 or CRHR2 has no effect on anxiety-like behavior in male rats under non-stress conditions (Sahuque et al., 2006).

Conversely, the F2 PNS females exhibited no overall decrease in the amount of time spent in the anxiogenic environments compared with the control females, similar to the findings in F1 females (Brunton and Russell, 2010); however in both the LDB and the EPM there was a significant influence of estrus cycle stage. F2 control females, displayed a significant reduction in anxiety-like behavior at proestrus/estrus, consistent with previous reports (Frye et al., 2000), however this effect was not observed in the F2 PNS females. Estradiol modulates anxiety-like behavior across the estrus cycle (Marcondes et al., 2001). Whether the differences in anxiety-like behavior at proestrus/estrus between F2 control and PNS rats reflects altered estradiol secretion or altered sensitivity to estradiol in F2 PNS females remains to be elucidated, though there is evidence from guinea pigs that prenatal stress is associated with lower plasma estradiol concentrations in the F1 offspring (Kapoor and Matthews, 2008). Reduced anxiety-like behavior at proestrus also coincides with increased circulating and central levels of the progesterone metabolite, allopregnanolone (Frye et al., 2000) an effect that can be abolished by treatment with the 5α-reductase (the rate-limiting enzyme responsible for converting progesterone into allopregnanolone) inhibitor, finasteride (Frye and Walf, 2002). Prenatal stress is associated with reduced 5α-reductase activity and gene expression in the brain (Paris et al., 2011), including in rats born to mothers exposed to social stress during pregnancy (Brunton et al., 2015). Thus, if a reduction in 5α-reductase activity and hence allopregnanolone production is also found in F2 PNS females, this may contribute to the absence of a reduction in anxiety-like behavior at proestrus/estrus. In contrast to the F2 males, we did not detect any differences in gene expression for *Crh* or the *Crh* receptors in the amygdaloid complex between F2 control and PNS females, consistent with our previous findings in F1 female offspring (Brunton et al., 2011, Brunton and Russell, 2010) and the behavior displayed at metestrus/diestrus (which accounts for ca. two thirds of the estrus cycle). However it is important to note that estrus cycle stage was not determined prior to culling, rather females were selected at random stages of the estrus cycle. Therefore we would predict differences in *Crh*, *Crhr1* and *Crhr2* mRNA expression may be detected between F2 control and PNS females if this were studied specifically at proestrus/estrus.

In humans, anxiety and depression are often comorbid (Kaufman and Charney, 2000). Moreover, several animal models of PNS report both depressive and anxious phenotypes (Poltyrev et al., 2005), hence we tested the F2 offspring for behavioral despair and anhedonia. We found no differences in these traits in either sex, indicating anxiety- and depression-like behaviors are differentially regulated in the F2 rats, and highlighting the potential to use this model to study anxiety-like behavior without the possible confounding effects of depressive-like traits.

The mechanisms through which maternal stress exerts a long-term impact on gene expression (i.e. *Gr*, *Mr*, *Crhr1* and *Crhr2* mRNA) in the offspring's brain are not known, though are likely to involve epigenetic changes e.g. altered DNA methylation of specific gene promoters (Bale, 2015), such as those described in the F1 offspring of mice exposed to stress during pregnancy (Mueller and Bale, 2008) or in the adult offspring of women exposed to extreme calorie restriction during pregnancy as a result of the Dutch famine (Heijmans et al., 2008). It is not known whether the transgenerational effects of prenatal stress in the F2 PNS offspring result from (i) maternal stress effects on the somatic cells of the F1 females (e.g. that may lead to an altered hormonal milieu or future maternal behaviour), that are then perpetuated in the F2 generation; (ii) a direct effect of maternal stress inducing epigenetic changes in the primordial germ cells of the F1 female (while *in utero*); or (iii) a combination of both. Heightened anxiety-like behavior in the F1 and F2 PNS males (together with similar changes in gene expression in the amygdala), but opposite effects of prenatal stress on HPA axis function in the F1 and F2 PNS males may indicate differential programming effects of maternal stress on the somatic cells and germ cells in the F1 offspring. Studies examining stress and anxiety phenotypes in the F3 offspring could help clarify the mechanism(s) involved (Bale, 2015).

In conclusion, the effects of grand-maternal social stress exposure during pregnancy on HPA axis regulation and anxiety-like behavior can be transferred via the maternal line to the second generation in a sex-specific manner. These findings have clinical implications for both the etiology of mood disorders and the management of pregnant women in order to promote healthy long-term outcomes for their children, and indeed future generations. Future research focusing on understanding the mechanisms underpinning transgenerational transmission of prenatal stress may facilitate the development of therapies and/or intervention strategies for affective disorders in humans.

## Funding

This work was supported by Biotechnology and Biological Sciences Research Council (BBSRC). The funding body had no role in the study design; the collection, analysis and interpretation of data; the writing of the report; and in the decision to submit the article for publication.

## Conflict of interest

The authors report no conflicts of interest.

## Author’s contributions

PB and NG were both involved in designing the studies, performing the experiments, analyzing the data and writing the paper.

## Figures and Tables

**Table 1:** F2 Litter characteristics.

Measure	Females			Males		
	F2 Control	F2 PNS	Statistics	F2 Control	F2 PNS	Statistics
Median number of pups	7 (IQR = 6-8.25)	8.5 (IQR = 8-9.75)	U(14) = 36.5, Z = 1.643, *p* = 0.11	4.5 (IQR = 3-6.25)	6 (IQR = 4.5-6.75)	U(14) = 32.5, Z = 1.134, *p* = 0.28
Mean birth weight (g)	6.48 ± 0.36	5.76 ± 0.19	t(12) = 1.886, *p* = 0.08	6.68 ± 0.31	6.04 ± 0.26	t(12) = 1.563, *p* = 0.14

The median number of pups of each sex/litter is given with the interquartile range (IQR) in parenthesis. Birth weight is given as mean ± SEM. Data are for the F2 litters born to F1 control dams (*n* = 6) and F1 PNS dams (*n* = 8).

**Table 2:** Behavior exhibited during the forced swim test.

Time spent (s)	FEMALES		MALES	
	F2 control	F2 PNS	F2 control	F2 PNS
Floating	117.3 ± 7.4	120.4 ± 6.9	202.7 ± 19.8	174.3 ± 13.5
Swimming	111.7 ± 11.3	101.8 ± 6.8	60.7 ± 17.1	65.7 ± 6.1
Climbing	66.8 ± 6.5	76.4 ± 6.7	33.5 ± 11.0	59.0 ± 11.0
Diving	4.3 ± 2.9	1.0 ± 0.5	3.3 ± 2.4	1.4 ± 0.7

Time spent (s) exhibiting different behaviors in the forced swim test. Data are group means ± SEM. *n* = 10 rats/group.
